# Ultrafast Excited-State Localization in Cs_2_AgBiBr_6_ Double Perovskite

**DOI:** 10.1021/acs.jpclett.1c00653

**Published:** 2021-03-30

**Authors:** Adam D. Wright, Leonardo R. V. Buizza, Kimberley J. Savill, Giulia Longo, Henry J. Snaith, Michael B. Johnston, Laura M. Herz

**Affiliations:** † Department of Physics, University of Oxford, Clarendon Laboratory, Parks Road, Oxford OX1 3PU, United Kingdom; ‡ TUM Institute for Advanced Study, Lichtenbergstraße 2a, 85748 Garching bei München, Germany

## Abstract

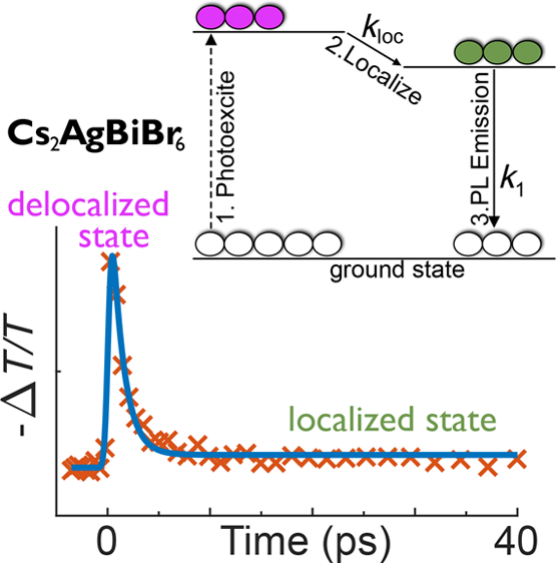

Cs_2_AgBiBr_6_ is a promising metal halide double perovskite offering the possibility of efficient photovoltaic devices based on lead-free materials. Here, we report on the evolution of photoexcited charge carriers in Cs_2_AgBiBr_6_ using a combination of temperature-dependent photoluminescence, absorption and optical pump–terahertz probe spectroscopy. We observe rapid decays in terahertz photoconductivity transients that reveal an ultrafast, barrier-free localization of free carriers on the time scale of 1.0 ps to an intrinsic small polaronic state. While the initially photogenerated delocalized charge carriers show bandlike transport, the self-trapped, small polaronic state exhibits temperature-activated mobilities, allowing the mobilities of both to still exceed 1 cm^2^ V^–1^ s^–1^ at room temperature. Self-trapped charge carriers subsequently diffuse to color centers, causing broad emission that is strongly red-shifted from a direct band edge whose band gap and associated exciton binding energy shrink with increasing temperature in a correlated manner. Overall, our observations suggest that strong electron–phonon coupling in this material induces rapid charge-carrier localization.

Metal halide perovskite materials have achieved remarkable success as photovoltaic active layers over the past decade,^1,2^ with the record power conversion efficiency (PCE) of perovskite solar cells now exceeding 25%.^3^ This success has resulted from the excellent optoelectronic properties of these materials, including their strong absorption across the visible spectrum and high charge-carrier mobilities.^1,4^ The presence of toxic lead in a readily water-soluble form in the principal perovskite photovoltaic materials, such as methylammonium lead triiodide (MAPbI_3_, MA = CH_3_NH_3_), is however a concern from a health and environmental perspective.^5^ Although attempts at homovalent replacement of the lead cation (Pb^2+^) by Ge^2+^ or Sn^2+^ have suffered from instability against oxidation,^6^ heterovalent substitution by monovalent and trivalent cations offers a larger compositional space of possible materials.^7^ Perhaps the most prominent^6^ of the resultant family of double perovskite materials is Cs_2_AgBiBr_6_, the extensive study of which has resulted in its successful fabrication in the form of single crystals,^8,9^ nanocrystals,^10^ and thin films through both solution-processing and vapor-deposition routes.^11,12^ Cs_2_AgBiBr_6_ has demonstrated a better thermodynamic stability than MAPbI_3_,^10^ but power conversion efficiencies in solar cells based on the former lag behind, having reached only 2.84% at best.^13^ The indirect band gap of this material is a hindrance to its photovoltaic performance,^14^ since the large film thicknesses thus required to substantially improve photocurrent generation would also necessitate very long charge-carrier diffusion lengths: of the order of tens of micrometers rather than the current tens of nanometers.^12^ Nonetheless, Cs_2_AgBiBr_6_ also possesses more positive optoelectronic properties for photovoltaic purposes, such as a long-lived component to the charge-carrier lifetime^9,15,16^ and a band gap that is relatively narrow for a double perovskite, being in the visible range.^6^ This material has also shown promise as a photocatalyst,^17^ in photodetectors,^18^ and in X-ray detectors.^19,20^

Understanding the nature of the coupling between the lattice and the charge carriers in Cs_2_AgBiBr_6_, and its influence on charge-carrier dynamics, is crucial for the optimization of this material in a variety of optoelectronic applications and to further the understanding of a wide range of related silver–bismuth–halide semiconducting materials.^21,22^ Such electron–phonon coupling may limit the charge-carrier mobility of a material, as is the case for MAPbI_3_.^1,23^ Because the electron–phonon coupling in Cs_2_AgBiBr_6_ is even stronger than in MAPbI_3_,^24−26^ it may potentially cause self-trapping of charge carriers. The phenomenon of self-trapping occurs when the local lattice distortion caused by a photoexcited charge carrier is sufficiently strong that the charge carrier rapidly relaxes into the energetic state associated with this local deformation,^27^ such that its localization length may approach the length of a single unit cell of the lattice.^28^ Self-trapping of charge carriers has been reported in related materials, whether for electrons in CsPbI_3_^29^ or for holes in CsPbBr_3_,^30^ in other bismuth-based materials such as Rb_4_Ag_2_BiBr_9_^31^ and Cs_3_Bi_2_Br_9_,^32^ and layered metal halide perovskites.^33^ For Cs_2_AgBiBr_6_, the proposed self-trapping^34^ has also been synonymously^35^ described as the formation of small polarons^24^ or color centers,^25^ with broad photoluminescence (PL) emission^25,34^ and low charge-carrier mobility^24^ attributed to its occurrence. The mobility of charge carriers has however alternatively been interpreted in terms of large polarons,^26^ which are shallow bound states of charge carriers associated with a lattice distortion extended over tens to hundreds of unit cells and result from weaker electron–phonon coupling.^28,36^ Proponents of this interpretation argue that the energetic barrier^37^ to small polaron formation in three-dimensional materials is too great.^26,37^ However, Cs_2_AgBiBr_6_ has in fact been described as having 0D electronic dimensionality because of the spatial isolation between the [AgBr_6_]^5–^ and [BiBr_6_]^3–^ octahedra that respectively determine its valence band maximum and conduction band minimum,^21,22,38,39^ which causes localization of photoexcited charge carriers^24,38^ and consequently high charge-carrier effective masses and low mobilities.^22^ Thus, in Cs_2_AgBiBr_6_ the existence of charge-carrier self-trapping to form small polarons remains a plausible proposal, but one that has not yet been either convincingly proven or directly observed.

In this work, we investigate the optoelectronic properties of Cs_2_AgBiBr_6_ and report the direct observation of ultrafast charge-carrier self-localization from an initially highly mobile delocalized state to a self-trapped, small polaronic state, which ultimately diffuses to an emitting color center. Through a combination of temperature-dependent PL and absorption spectroscopy, we can identify the electronic states occupied before and after localization and characterize their relation to the direct and indirect gap transitions in this material. The localization of charge carriers with time is directly traced by using PL upconversion spectroscopy and optical pump terahertz probe (OPTP) measurements, and we interpret its influence on the charge-carrier mobility in terms of a quantitative model that allows us to identify a temperature-invariant localization rate of 0.99 ps^–1^. We therefore conclude that the electron–phonon coupling in this material is sufficiently strong to lead to the formation of intrinsically self-trapped charge carriers and consider the consequent implications on the application of Cs_2_AgBiBr_6_ and other double perovskites in photovoltaic and other optoelectronic devices.

To explore the nature of the electronic states in Cs_2_AgBiBr_6_, we first measured the temperature dependence of the absorption and PL spectra of vapor-deposited thin films of this material between 4 and 295 K (see sections 1 and 2 of the Supporting Information for details of the sample fabrication and experimental setup). The spectra measured at 4 and 295 K are shown in Figures 1a and 1b, respectively. At room temperature, the PL spectrum peaks at 1.95 eV, in line with previous studies.^9,24,25,40^ Cs_2_AgBiBr_6_ has been reported to be an indirect band gap semiconductor,^6,8,25,41^ and this PL emission has in the past been assigned to phonon-assisted recombination across the indirect band gap;^8,9,24,41^ however, more recent PL excitation measurements have indicated that it may instead result from spatially localized color centers.^25^ Meanwhile, previous calculations^38,41−44^ and experimental measurements^8−10,12,16,24,25,34,40,44,45^ have reported the room temperature indirect gap to lie in the range 1.79–2.25 eV. This range is also consistent with the value of 1.9 eV obtained by our quadratic fit to the absorption spectrum at energies below the direct gap, as detailed in section 3.1 of the Supporting Information. The temperature dependence of the PL spectrum is shown in Figure 1c, while that of its peak energy is compared with the indirect gap energy in Figure 2b. The position of the PL peak at energies above the indirect gap energy, and their opposite trends in energy shifts with temperature, suggests that the PL does not originate from a band-to-band transition across the indirect gap but is rather linked to a different transition, albeit with a similar energy, involving bound excitons localized at a color center.^25,42^ A bound exciton refers to a Coulombically bound electron–hole pair which is localized to a particular point on the lattice. The ≈20 meV red-shift of the PL peak between 4 and 295 K may result from the enhanced relaxation of charge carriers between color-center states, whose energetic spread is indicated by the red-shift. At higher temperatures, more thermal energy is available for the charge carriers to activate over the energetic barriers between these states, which could allow them to reach deeper color centers before recombining.^46^

**Figure 1:**
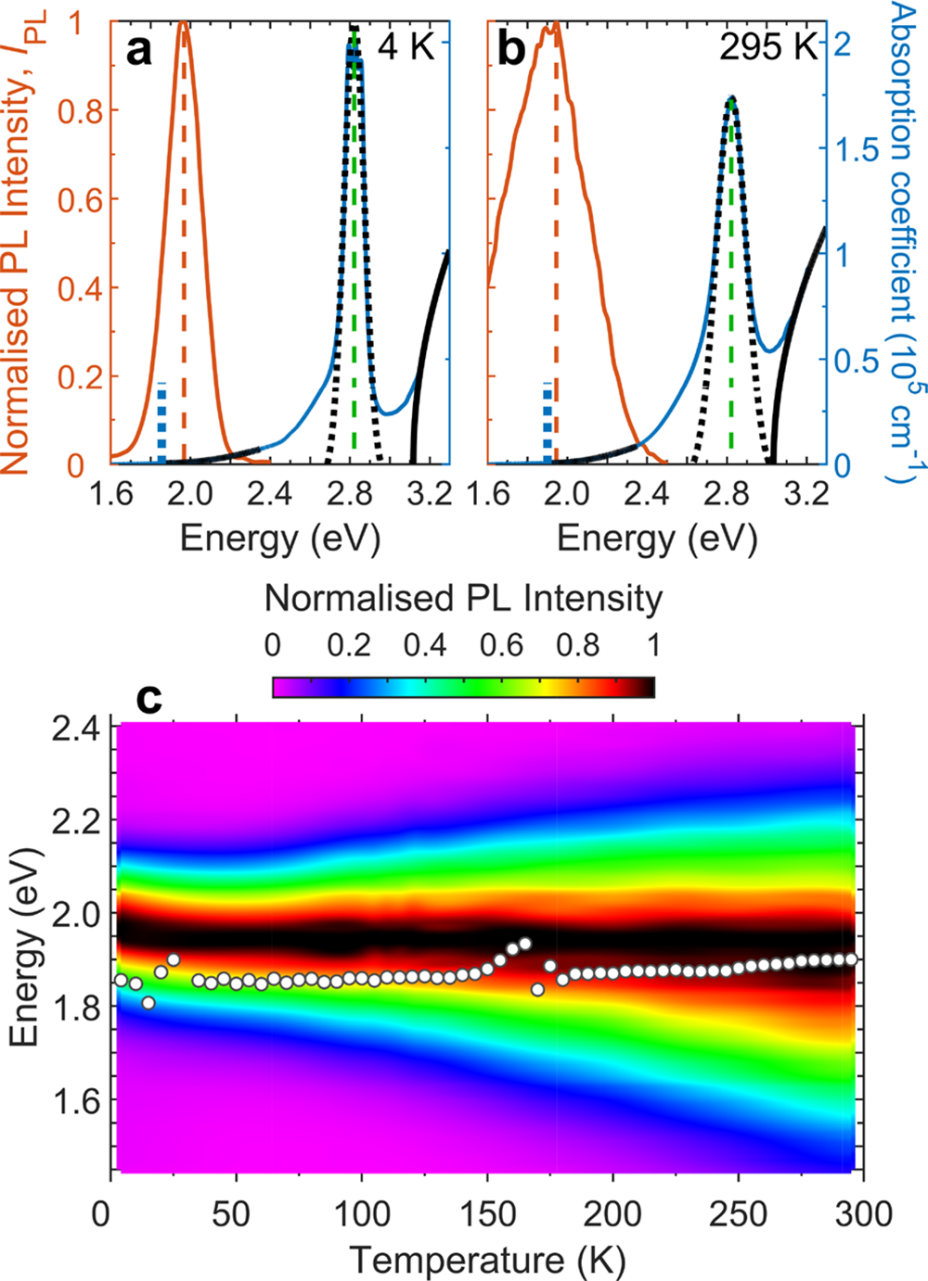
Temperature-dependent absorption and PL spectra of Cs_2_AgBiBr_6_. (a) Steady-state PL (solid red line) and absorption spectra (solid blue line) at 4 K as a function of photon energy for a Cs_2_AgBiBr_6_ thin film. A Gaussian fit to the excitonic absorption peak at around 2.8 eV is plotted as a dotted black line, with its central energy corresponding to the dashed green line. The dashed red line indicates the energy at the maximum PL intensity. Quadratic and square-root fits to the lower-energy indirect absorption onset and higher-energy direct absorption onset respectively are plotted as solid black lines. Close-up views of the absorption spectrum in the regions of the fits are shown in Figure S1. The dotted blue line indicates *E*_g_^i^, which provides an estimate of the indirect band gap energy from the quadratic fit. (b) Corresponding spectra and fits at 295 K. (c) Color plot of the normalized PL spectra at temperatures between 4 and 295 K. The white circles show *E*_g_^i^ as a function of temperature.

**Figure 2:**
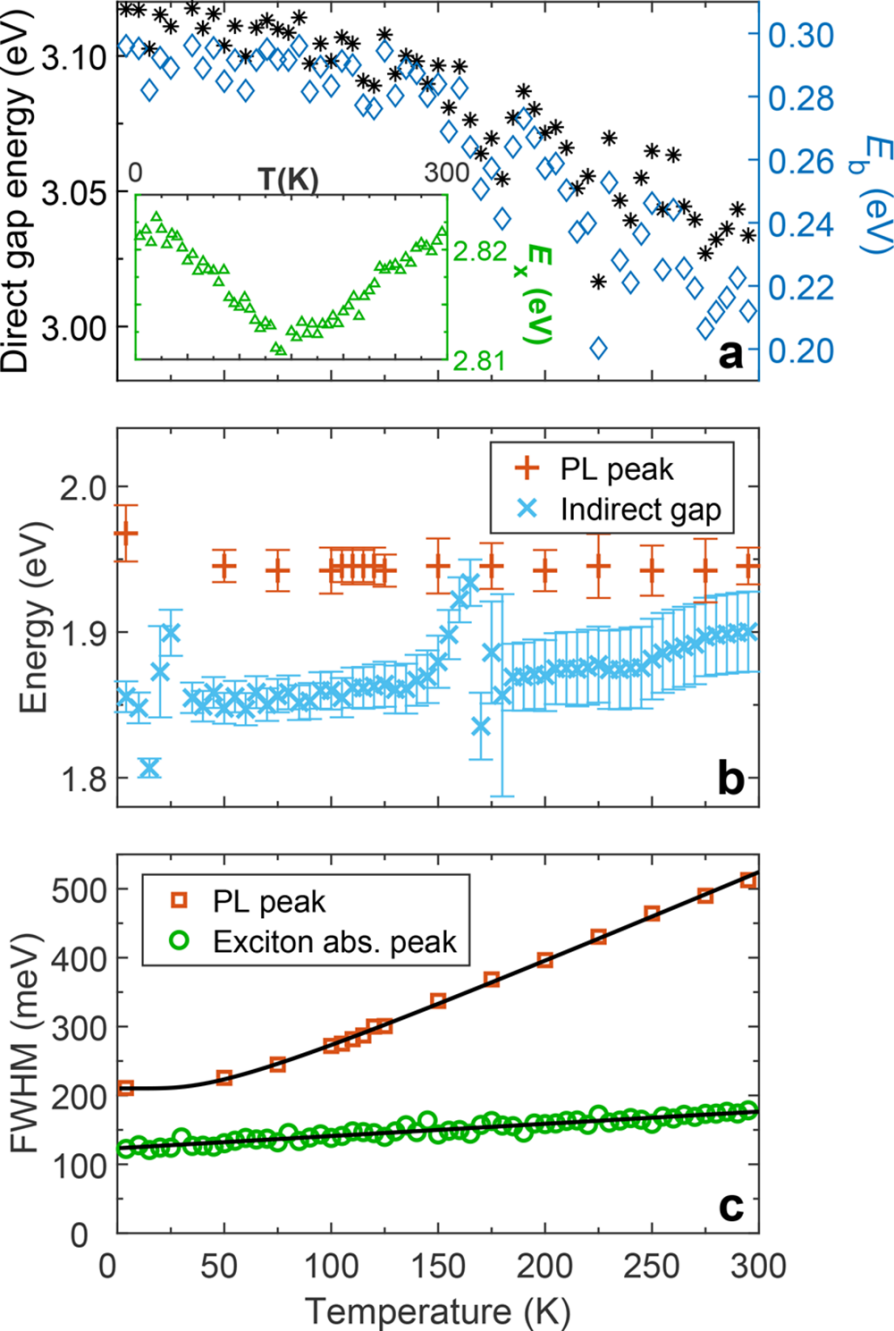
Temperature-dependence of transition energies and peak widths of Cs_2_AgBiBr_6_. (a) Direct band gap energy (black asterisks, left axis) and direct exciton binding energy (*E*_b_, blue diamonds, right axis) of Cs_2_AgBiBr_6_ as a function of temperature, as obtained from fits to absorption spectra. The inset shows the temperature dependence of the direct exciton energy (*E*_x_, green triangles), obtained from Gaussian fits to the peak evident in absorption spectra. (b) Temperature dependence of the peak of the PL spectra (red plus signs) and the indirect band gap energy (light blue crosses) as determined from the absorption spectra. (c) Temperature-dependent line width (full width at half-maximum, FWHM) of the PL peak (red squares) and excitonic absorption peak (green circles). The solid black lines are fits that account for Fröhlich coupling with LO phonons, as described in the main text.

The absorption spectra in Figures 1a and 1b are dominated by a strongly absorbing feature at ∼2.8 eV, which has usually been described as an excitonic peak,^9,25,34,47^ although alternative explanations have attributed it to one or more transitions between atomic orbitals either within the Bi^3+^ ion or involving the Ag^+^ ion as well.^10,48,49^ Attributing an excitonic nature to the absorption peak has faced contentions that the resultant exciton line width and binding energy values are too large for what should be expected of Cs_2_AgBiBr_6_.^48,49^ However, the resonance in the reflectance spectrum of Cs_2_AgBiBr_6_ at 2.8 eV is characteristic of a band-edge, free-exciton absorption,^9,25^ while Kentsch et al.^34^ noted that similar excitonic absorption features are common features of bismuth-containing perovskites and their relatives and found evidence from Raman and transient absorption spectroscopy for a considerable excitonic contribution to the feature in Cs_2_AgBiBr_6_. Furthermore, Palummo et al.^42^ were able to ascribe the absorption peak to a bound direct exciton using *ab initio* methods, noting that the large exciton binding energy compared to MAPbI_3_ could be explained by the reduced ionic contribution to dielectric screening in Cs_2_AgBiBr_6_. Dey et al.^50^ also attribute this peak to a bound exciton, whereas Lv et al.^51^ attribute it to a free exciton, i.e., a Coulombically bound electron–hole pair which may move freely throughout the lattice. A further objection to describing the absorption peak as excitonic is based on the apparent invariance of its energy with temperature,^10,48^ which would not be expected behavior for an excitonic emission that would normally shift with temperature according to the band gap trend. However, as we reveal below, the absorption peak energy actually varies slightly with temperature, but with the magnitude of the shift being reduced because the associated exciton binding energy shifts with temperature in close correlation with the direct band gap energy. We therefore consider this absorption feature to be an excitonic peak and fit it with a Gaussian function to parametrize its central energy and line width.

The temperature dependence of the excitonic absorption peak line width is compared with that of the PL peak in Figure 2c, in which both data sets are quantitatively assessed by using the function^23^ Γ(*T*) = Γ_0_ + Γ_LO_, where Γ_LO_ = γ_LO_/(e^*E*_LO_/*k*_B_*T*^ – 1) , which is commonly employed to describe the temperature dependence of the PL or absorption line width. Here, Γ_0_ is a temperature-independent inhomogeneous scattering term arising from disorder and imperfections, whereas the temperature-dependent homogeneous scattering from longitudinal optical (LO) phonons via the Fröhlich interaction contributes with charge-carrier coupling strength γ_LO_ for a representative LO phonon energy *E*_LO_.^52^ Fits of Γ(*T*) (solid black lines in Figure 2c) to the excitonic absorption and PL peak line widths describe the data well, resulting in output parameters of Γ_0_ = 123 meV, γ_LO_ = 0.12 meV, and *E*_LO_ = 0.06 meV for the excitonic peak and Γ_0_ = 210 meV, γ_LO_ = 175 meV, and *E*_LO_ = 11.5 meV for the PL peak. Even without accounting for the acoustic phonon contribution to homogeneous scattering, it is clear that the PL peak broadens much more with temperature than does the direct gap excitonic peak, perhaps indicative of a stronger coupling to the lattice by the self-trapped state responsible for the former compared with the direct excitons responsible for the latter. The LO phonon coupling strength and energy obtained from the PL peak are comparable to those previously reported,^24^ with the coupling strength several times that of MAPbI_3_ (for which γ_LO_ = 40 meV),^23^ in agreement with the strong electron–phonon coupling that has been attributed to Cs_2_AgBiBr_6_^24−26^ and which is likely to be responsible for the self-localization of the excitons therein.

Figure 2a shows the temperature dependence of the direct gap *E*_g_, obtained from a square-root fit to the absorption spectrum as detailed in section 3.1 of the Supporting Information. The direct gap energy red-shifts from 3.12 to 3.03 eV between 4 and 295 K, which is unlike the corresponding blue-shift of the direct gap in MAPbI_3_ but is, however, in accordance with the typical behavior of semiconductors such as Si, Ge, and GaAs.^53^ Tauc plots to absorption spectra have often underestimated the room-temperature direct gap of Cs_2_AgBiBr_6_ by several hundred millielectronvolts^8,24,54^ by placing it between 2.2 and 2.41 eV, possibly as a result of mistaking the excitonic peak for the direct gap onset. There have nonetheless also been reports placing the direct gap energy in the range 2.85–3.20 eV at 295 K,^9,34,48^ which is consistent with our measured value. The direct exciton binding energy *E*_b_ is also plotted in Figure 2a, obtained by subtracting the central energy of the Gaussian fit to the excitonic absorption peak (plotted in the inset to Figure 2a) from the direct gap energy. In section 3.2 of the Supporting Information we alternatively apply a fit based on Elliott’s theory^55^ to the direct gap onset to obtain *E*_b_ by a more sophisticated method but find that the overlap between the excitonic and continuum contributions to the absorption is sufficiently small that our original approach of obtaining the excitonic and direct gap energies separately is in excellent agreement with the Elliott fitting approach. We find that *E*_b_ also decreases as the temperature increases from 4 to 295 K, reaching 210 meV at room temperature, which is slightly smaller than previous estimates that have placed it between 268 and 340 meV.^34,42^ As mentioned above, such values of *E*_b_ are relatively large compared to MAPbI_3_ (for which *E*_b_ ∼ 10 meV^4,56^) but are typical for similar bismuth-based materials.^34^ We note that the prominence of the direct excitonic absorption peak at room temperature in Cs_2_AgBiBr_6_ compared to MAPbI_3_ can be attributed to the much larger value of *E*_b_ for the former. The apparent positive correlation between the temperature dependences of *E*_b_ and the direct gap energy in Cs_2_AgBiBr_6_ is an example of a relationship that has been empirically shown to hold more generally for 3D semiconductors.^57,58^ Such a correlation ultimately derives from both band gap energies and exciton binding energies being dependent on factors such as effective charge-carrier masses and values of the dielectric function, which may vary for example with material composition or temperature. We note that unlike *E*_b_, the excitonic absorption peak energy (*E*_x_ = *E*_g_ – *E*_b_, plotted in the inset to Figure 2a) shows only minor changes with temperature, of the order of 10 meV, largely because the temperature dependences of *E*_g_ and *E*_b_ almost completely cancel. However, within these minor shifts the excitonic absorption peak energy *E*_x_ is seen to undergo a reversal in its temperature trend at around 130 K, roughly corresponding to the phase transition between the low-temperature tetragonal phase and the high-temperature cubic phase.^9^ These changes in excitonic peak position *E*_x_ around the phase transition may result from a corresponding shift in the dielectric constant^9^ affecting the band gap and exciton binding energy to a slightly different extent. In any case, the observed positive correlation between the exciton binding energy *E*_b_ and the direct gap energy supports our interpretation that the prominent absorption peak arises from a direct exciton.

Having established the nature of the electronic states in Cs_2_AgBiBr_6_, we investigated the charge-carrier dynamics and mobility therein. Figure 3a shows temperature-dependent OPTP photoconductivity transients (plotted as the photoinduced transmission change −Δ*T*/*T* ) measured out to 40 ps after excitation, under the experimental conditions detailed in section 2.4 of the Supporting Information. The sample was excited at 400 nm (3.1 eV), which corresponds to a transition above the direct band gap. As shown in section 5.3 of the Supporting Information, the photoconductivity spectra across the measured energy range (0.5–10 meV) are Drude-like, in accordance with the presence of free charge carriers. The photon-to-charge branching ratio is thus likely to be close to 1, though we cannot rule out the formation of free excitons by the initial excitation. As discussed in section 5.3 of the Supporting Information, the high *E*_b_ of this material means that any intraexcitonic transitions would in any case be too large to be probed by our OPTP spectroscopy.

**Figure 3:**
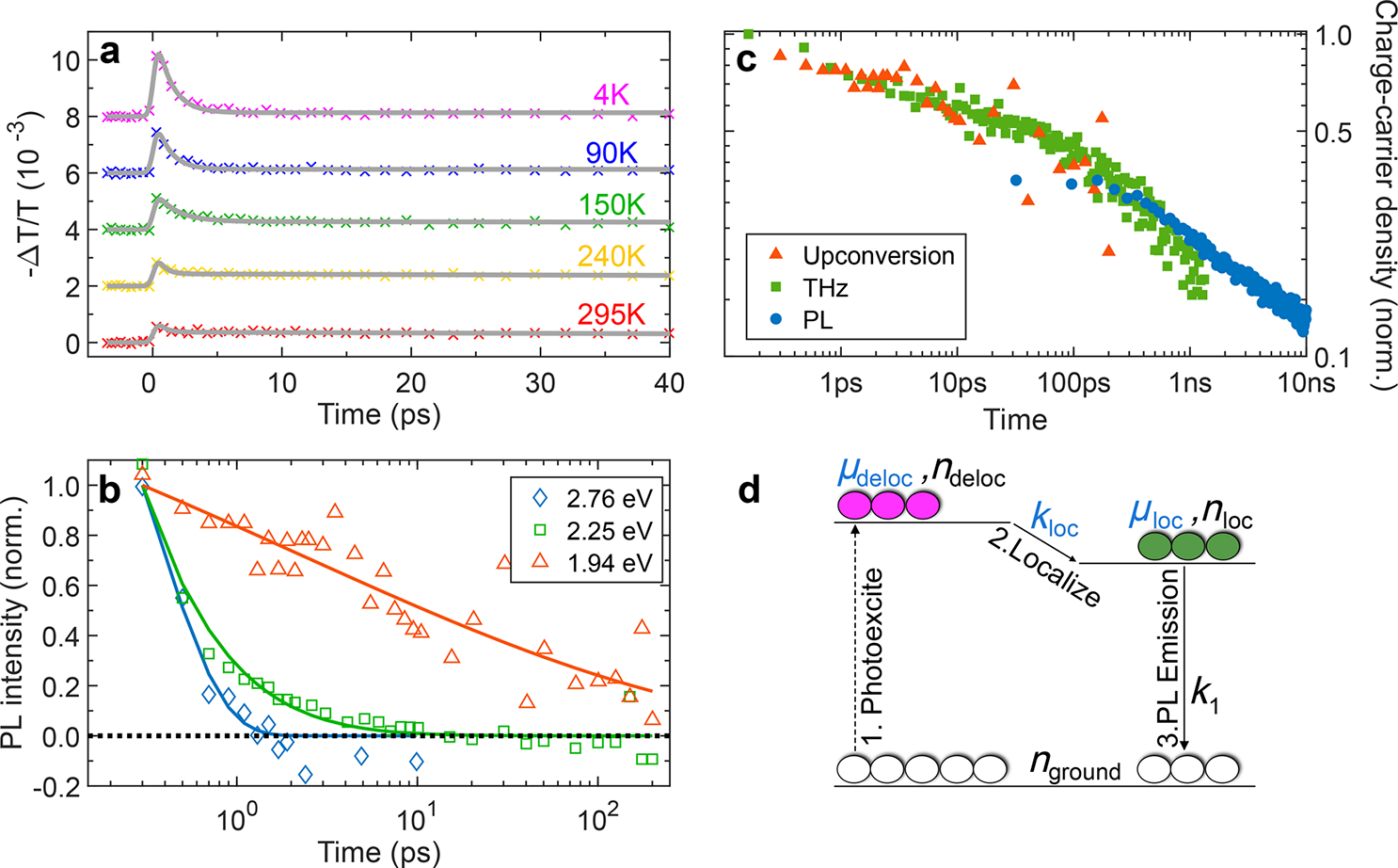
Dynamics of charge-carrier localization in Cs_2_AgBiBr_6_. (a) OPTP photoconductivity transients for a Cs_2_AgBiBr_6_ thin film, measured at the temperatures shown in the legend under an excitation fluence of 10.1 μ J cm^–2^. For clarity, transients at successively decreasing temperatures are offset vertically by 2 × 10^–3^. The gray lines depict fits based on a two-level charge-carrier mobility model, as described in the main text. (b) Room-temperature normalized PL upconversion transients measured at 2.76 eV (blue diamonds), 2.25 eV (green squares), and 1.94 eV (red triangles). The solid lines are stretched exponential fits to the transients (empty markers) of the corresponding colors. The dotted black line indicates zero intensity; negative intensities are an artifact of background subtraction. (c) Time dependence of charge-carrier density, *n*, after photoexcitation, as derived from OPTP photoconductivity (green squares), PL upconversion (red triangles), and TCSPC (blue circles) measurements. The latter two transients are scaled to line up with the terahertz transient, which is normalized at its maximum intensity point. (d) Schematic of the two-level mobility model used to fit the OPTP decays in (a). The fixed parameters are shown in black, while parameters that are fitted and extracted from the model are shown in blue. After being initially photoexcited to the delocalized state (pink), the charge carriers localize to the localized state (green) before recombining.

A fast decay component is evident in all the photoconductivity transients, though more prominent at lower temperatures, followed by a long-lived decay. Such behavior is qualitatively consistent with the fast localization of charge carriers from an initial state with high mobility (reflected in the value of −Δ*T*/*T* at the peak of the transient) to a state whose lower mobility is indicated by the lower value of −Δ*T*/*T* on longer time scales. Localization is also evident in Figure 3b, in which room-temperature ultrafast PL transients measured at emission wavelengths of 2.76, 2.25, and 1.94 eV (using PL upconversion^59−61^ as described in section 2.5 of the Supporting Information) are plotted, following the charge carriers as they rapidly shift downward in energy over subpicosecond time scales to progressively longer-lived states: the transients for the three excitation energies were phenomenologically parametrized by average lifetimes of 0.34, 0.65, and 340 ps, respectively, obtained by fitting stretched exponential functions. The short-lived, high-energy PL transient measured at 2.76 eV corresponds to the direct exciton state identified in the absorption spectrum shown in Figure 1b, while the longer-lived, low-energy transient measured at 1.94 eV results from the principal PL emission also plotted in that figure and which was attributed to a color center.^25^ Meanwhile, the transient measured at 2.25 eV captures the emission from excitons at an intermediate stage between the direct exciton and color center states. Because PL derives from the bimolecular recombination of free carriers, or the recombination of excitons formed in bimolecular fashion from free carriers, its intensity is directly proportional to *n*^2^, whereas the −Δ*T*/*T* in OPTP transients is directly proportional to *n*. By plotting the normalized charge-carrier densities, *n*, derived from the 1.94 eV PL upconversion transient and the 295 K OPTP transient on the same graph (Figure 3c), their overlap demonstrates that the charge-carrier behavior captured by the two techniques is consistent, with the recombination of the charge carriers at the principal PL peak^48^ driving the decay in the total charge-carrier density measured by OPTP spectroscopy, once the initial localization has taken place. Using PL measured at 1.95 eV by time-correlated single photon counting (TCSPC), the time dependence of *n* is extended to later times, though this technique lacks the time resolution to capture the initial fast localization of charge carriers. The temperature-dependent dynamics of the TCSPC transients are presented in section 4 of the Supporting Information, extending to much longer time scales than the PL upconversion transients and thus providing more meaningful estimates of the PL lifetime. Although charge-carrier dynamics in metal halide perovskites often depend on how the sample was processed,^62^ the shape of these transients is in fact similar to those measured for single crystals of Cs_2_AgBiBr_6_,^9,40^ which suggests that charge-carrier recombination in this material is dominated by intrinsic factors. In combination, the three measurement techniques follow the charge carriers as they localize over ultrafast time scales from an incipient delocalized state to more localized states that are downshifted in energy.

To quantitatively interpret the OPTP terahertz photoconductivity transients in Figure 3a in terms of localization of charge carriers upon transitioning from one state to another, we developed a simple two-level model of the early time charge-carrier dynamics (depicted in Figure 3d and described in detail in section 5.1 of the Supporting Information) which was used to fit the transients. In brief, the model allows for two excited states of the charge carriers: a delocalized state associated with a high mobility, μ_deloc_, and a localized state associated with a low mobility, μ_loc_. Photoexcitation occurs into the delocalized state, from which charge carriers may localize at rate *k*_loc_ to the localized state. The initial delocalized state determines the initial terahertz photoconductivity response, while its ultrafast emission was evident in the PL upconversion transient measured at 2.76 eV in Figure 3b. Charge carriers in the localized state then diffuse to color centers and recombine to the ground state with recombination rate *k*_1_, producing the PL peak which we observed in Figure 1c and the TCSPC transients in Figure S3, the lifetimes of which we used to estimate *k*_1_. By assuming that only these transitions between states are permitted, we can describe the time evolution of the charge-carrier density in each state in terms of the aforementioned rate constants and hence relate these densities to the measured −Δ*T*/*T* in the OPTP transients via the sheet conductivity, which is given by
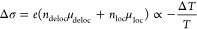
1We find that the early time charge-carrier dynamics of Cs_2_AgBiBr_6_ can indeed be well-described by a two-state localization model, as evidenced by the good fits of the resultant model function to the OPTP transients in Figure 3a. Such fits to the temperature-dependent transients yield the corresponding localization rate and charge-carrier mobilities, which are respectively plotted in Figures 4a and 4b. At low temperatures, the transients rapidly decay almost to zero as the localized state becomes populated, indicating that its mobility (≈ 0.5 cm^2^ V^–1^ s^–1^) is far lower than the charge-carrier mobility of the initial delocalized state (≈ 12 cm^2^ V^–1^ s^–1^). In contrast, the high-temperature transients do not drop as sharply, possessing a long tail which indicates an increased charge-carrier mobility (≈ 1.3 cm^2^ V^–1^ s^–1^ at 295 K) of the localized state at higher temperatures, approaching that of the delocalized state (≈ 3 cm^2^ V^–1^ s^–1^ at 295 K). It is thus apparent that the difference between the mobilities of the delocalized and localized states becomes smaller with increasing temperature, as quantified in Figure 4b, which shows the mobilities obtained from the two-state model. Notably, the localization rate seems largely temperature-independent, with a mean value of *k*_loc_ = 0.99 ± 0.43 ps^–1^ across all temperatures, suggesting that the process is barrier-free rather than temperature-activated. In contrast, the mobilities of both states vary with temperature, with that of the delocalized state decreasing at higher temperatures, while the localized state mobility exhibits the opposite trend. These trends may be quantified by fitting a power-law dependence, μ ∝ *T*^*p*^ to the data, yielding exponents of *p* = −0.47 ± 0.13 for μ_deloc_ and *p* = 0.64 ± 0.32 for μ_loc_. For the delocalized state, we neglect the two lowest temperature data points, since the power-law model diverges unphysically in this region.^63^ The temperature dependence of charge-carrier mobility is strongly influenced by the type of lattice coupling or scattering experienced by charge carriers, with potentially multiple contributions involved.^64,65^ As discussed further below, the negative exponent for μ_deloc_ clearly suggests bandlike transport as expected for delocalized charge carriers, while the positive exponent for μ_loc_ is commensurate with the temperature-activated hopping transport associated with localized, self-trapped states.^66^

**Figure 4:**
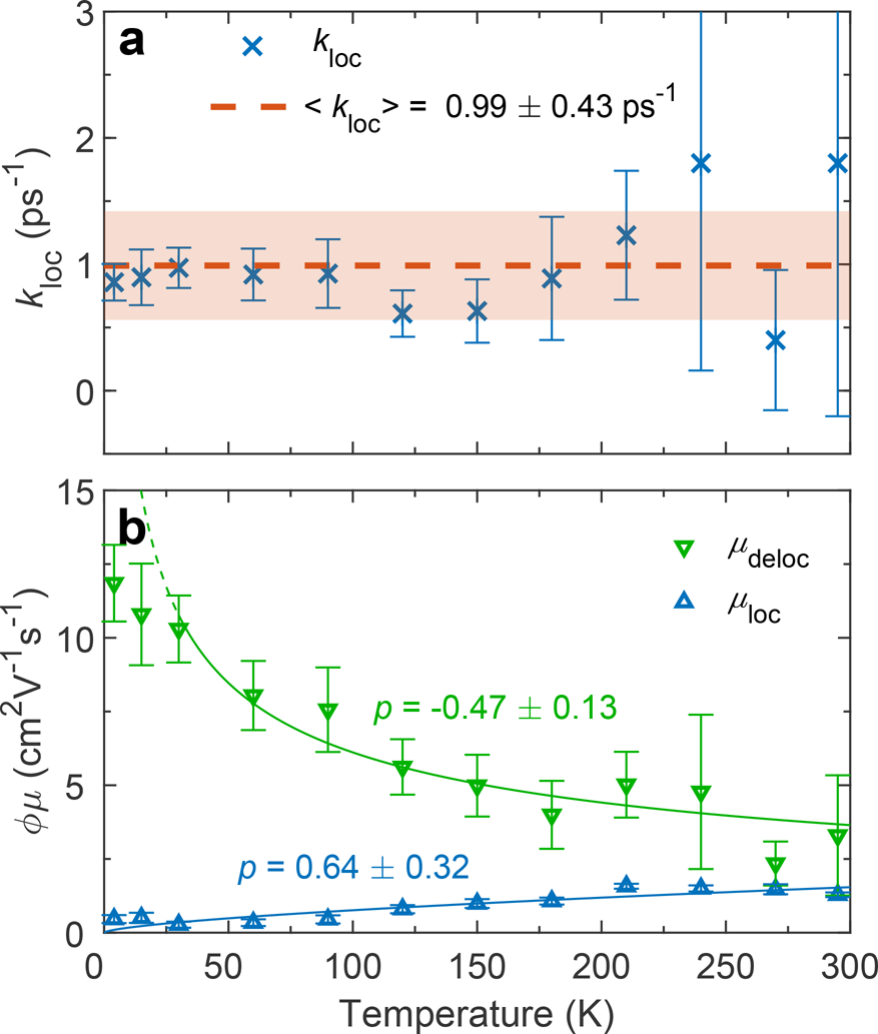
Charge-carrier mobility changes indicative of excited-state localization. (a) Temperature dependence of the localization rate (*k*_loc_, blue crosses) within the two-state mobility model for Cs_2_AgBiBr_6_. The red dashed line indicates the mean value, with its uncertainty represented by the red shaded area. (b) Temperature dependence of the effective charge-carrier mobilities associated with the delocalized (μ_deloc_, green) and localized (μ_loc_, blue) states. Power-law fits are plotted as solid lines in the corresponding colors, with their exponents (*p*) displayed alongside. The data points for μ_deloc_ at the lowest two temperatures were not included in the fit as the power-law model becomes unphysical in this region, indicated by the dashed green line. The mobilities are “effective” since the photon-to-charge branching ratio, ϕ, is not necessarily 1.

Overall, our detailed investigation based on multiple complementary techniques and across a wide temperature range allows us to provide a holistic explanation of the electronic states and charge-carrier dynamics in Cs_2_AgBiBr_6_. Our analysis of the OPTP transients strongly supports the notion that a fast charge-carrier localization process exists in this material, through which charge carriers transition from an initially excited delocalized state to a subsequent self-trapped state. The mobility of the initial delocalized state varies with temperature according to a power law with *p* = −0.47, an exponent which is not as negative as that commonly measured for MAPbI_3_,^62^ but whose sign is still indicative of bandlike transport limited by phonon scattering.^63,67^ We associate this state with the direct band gap (of 3.03 eV at 295 K) observed in the absorption spectra. From the OPTP transients, we detected a lower-energy state to which the charge-carriers relax, whose mobility varies with a positive exponent, *p* = 0.64, which is highly indicative of the temperature-activated hopping transport typical of localized charge carriers.^68,69^ The low energetic barrier to the formation of this localized state (indicated by the temperature invariance of the localization rate) is consistent with the formation of intrinsically self-trapped excitons, which have also been observed to form in a quasi-barrierless manner in 2D metal halide perovskites.^70^ We thus propose that this localized state is a self-trapped or small polaronic state, formed through an intrinsic process involving relaxation of the lattice around the excited state, rather than mediated by defects, given that localization is ultrafast and temperature-independent. This picture is consistent with the strong electron–phonon coupling we observe in Cs_2_AgBiBr_6_. This self-trapped state is clearly still mobile, but its motion is akin to that of a small polaron, which carries significant lattice distortion with it and therefore has a temperature-activated mobility.

We note that the self-trapped state we observe to form on a picosecond time scale is unlikely to be the final state leading to the principal emission observed from Cs_2_AgBiBr_6_ near 1.95 eV at room temperature. As discussed above, we confirm that the lack of correlation between the temperature dependence of this PL peak with the lower-energy indirect band gap means that the PL emission does not result from band-to-band transitions across the indirect gap but rather from a color center. Such color-center emission is also consistent with our observations of strong electron–phonon coupling in the temperature-dependent PL broadening of Cs_2_AgBiBr_6_ and a large energetic shift of the PL peak from the direct gap. However, the ultrafast initial localization step we observe in the photoconductivity transients is unlikely to be associated with the trapping of charge carriers at color centers, for the following reasons. First, the self-trapped state still has a respectable associated mobility of ≈1.3 cm^2^ V^–1^ s^–1^ at 295 K, which cannot originate from color centers that trap charge carriers relatively tightly.^27^ Second, the relaxation to the self-trapped state is ultrafast, meaning that the density of color centers would have to be incredibly high, which is unlikely given that our Cs_2_AgBiBr_6_ thin films are highly crystalline.^12^ Finally, the relaxation rate is also temperature-independent, which is inconsistent with diffusion of charge carriers to color centers.

Altogether, we report evidence for ultrafast localization of charge carriers in the Cs_2_AgBiBr_6_ double perovskite. We observed rapid decays in OPTP transients, which we were able to interpret in terms of a two-state model involving relaxation of free carriers to a localized state on the time scale of 1.0 ps. By combining temperature-dependent PL and absorption measurements, we were able to interpret the localized state as intrinsically self-trapped, or small polaronic, in nature. Such self-trapped charge carriers subsequently diffuse to color centers that account for the broad and strongly red-shifted emission. Because this material is the flag bearer for its class, developing our understanding of its electronic transitions and charge-carrier dynamics is helpful in guiding the search for better such materials or optimizing Cs_2_AgBiBr_6_ for such applications as in solar cells or X-ray detectors. The finding of intrinsic self-trapping of charge carriers in this material may intuitively suggest that Cs_2_AgBiBr_6_ is not an ideal material to choose for optoelectronic devices that rely on high charge-carrier mobilities, even if all defects were eliminated. However, we note that such self-trapping is less severe at room temperature because temperature-activated hopping processes lead to a relatively benign reduction in charge-carrier mobility, with the mobilities of both delocalized and localized states exceeding a respectable 1 cm^2^ V^–1^ s^–1^. Thus, while intrinsic self-trapping is clearly present in Cs_2_AgBiBr_6_, temperature activation of charge motion at room temperature still renders this material a promising candidate for lead-free photovoltaic applications. In addition, self-trapping effects may potentially be tuned through factors such as electronic dimensionality and composition,^66^ which opens up prospects of optimized material design through further computational simulation and optoelectronic characterization.

## Supporting Information Available

Experimental details and sample fabrication methods, temperature-dependent photoluminescence transients, terahertz photoconductivity and dark conductivity spectra, details of fits to the absorption spectra and terahertz photoconductivity traces (PDF)
