# Supplementary material for: Ultrafast Excited-State Localization in Cs2AgBiBr6 Double Perovskite
Source: J Phys Chem Lett. 2021 Mar 30;12(13):3352–60. doi: 10.1021/acs.jpclett.1c00653 (PMC8154850; doi:10.1021/acs.jpclett.1c00653)
Supplement: Supplementary file 1 — jz1c00653_si_001.pdf [file jz1c00653_si_001.pdf]

Supporting Information: Ultrafast Excited-State Localization in $Cs_2AgBiBr_6$ Double Perovskite

Adam D. Wright[1], *Leonardo R. V. Buizza*[1], *Kimberley J. Savill*[1], *Giulia Longo*[1,2], *Henry J. Snaith*[1] *Michael B. Johnston*[1] *and Laura M. Herz*[1,3]*

Email: laura.herz@physics.ox.ac.uk

[1]Department of Physics, University of Oxford, Clarendon Laboratory, Parks Road, Oxford OX1 3PU, United Kingdom

[2] Department of Mathematics, Physics and Electrical Engineering, Northumbria University, Newcastle upon Tyne NE1 8ST, United Kingdom

[3] TUM Institute for Advanced Study, Lichtenbergstraße 2a, 85748 Garching bei München, Germany

Contents

1. Sample fabrication and characterization 2
2. Spectroscopic techniques 2
   2.1. Absorption spectroscopy 2
   2.2. iCCD photoluminescence spectroscopy 2
   2.3. Time-correlated single-photon counting (TCSPC) 2
   2.4. Optical pump terahertz probe (OPTP) spectroscopy 3
   2.5. Photoluminescence upconversion spectroscopy 3
3. Fits to the absorption spectra 4
   3.1. Indirect and direct band gap fits 4
   3.2. Elliott fitting 5
4. Temperature-dependent TCSPC measurements 7
5. THz photoconductivity 9
   5.1. Fitting of temperature-dependent decays 9
   5.2. Fluence-dependent OPTP THz photoconductivity spectroscopy transients 11
   5.3. OPTP THz photoconductivity spectra 12
   5.4. OPTP THz dark conductivity spectra 12

References 14

1. Sample fabrication and characterization

$Cs_2AgBiBr_6$ thin films were prepared through sequential vapour deposition onto z-cut quartz substrates, as reported previously.[1] The precursors were AgBr (99% purity, purchased from Fluka), $BiBr_3$ (≥ 98% purity, purchased from Sigma Aldrich) and CsBr (99.9% purity, purchased from Sigma Aldrich). These precursors were placed in separate crucibles within a sealed vacuum chamber, and sequentially thermally evaporated onto the substrates. The optimized procedure involved the evaporation of 90 nm of AgBr, 120 nm of $BiBr_3$ and 150nm of CsBr to obtain $Cs_2AgBiBr_6$ films of 290 nm thickness. The deposited films were annealed on a hotplate in air at 250 °C for 15 minutes to form the desired double perovskite phase of $Cs_2AgBiBr_6$. The successful formation of $Cs_2AgBiBr_6$ thin films by this procedure was confirmed by X-ray diffraction (XRD) of thin films deposited on FTO. The XRD pattern[1] exhibited sharp peaks (indicating high crystallinity) and confirmed the absence of any additional phases or unreacted precursors. As detailed in the main text, the absorption and photoluminescence spectra measured for our thin films on quartz substrates are also consistent with previous reports[1–3] while our measured dark photoconductivity spectrum (Figure S7) is in agreement with previous calculations[4] of the phonon modes of the $Cs_2AgBiBr_6$ lattice.

2. Spectroscopic techniques

2.1. Absorption spectroscopy

To measure the temperature-dependent absorption spectra of $Cs_2AgBiBr_6$ thin films, a Bruker Vertex 80v Fourier transform infrared (FTIR) spectrometer was used in both transmission and reflection geometry, configured with a tungsten halogen lamp illumination source, a CaF_2 beamsplitter and a silicon detector. The sample was mounted in a gas-exchange helium cryostat (Oxford Instruments, OptistatCF2) which allowed the temperature to be varied from 4 to 295 K in increments of between 5 and 10 K.

2.2. iCCD photoluminescence spectroscopy

To measure the temperature-dependent steady-state photoluminescence (PL) of $Cs_2AgBiBr_6$ thin films, the sample was photoexcited by a 398 nm diode laser (PicoHarp, LDH-D-C-405M). Continuous wave excitation with an intensity of 67 W cm^{-2} was used. The resultant PL was collected and coupled into a grating spectrometer (Princeton Instruments, SP-2558), which directed the spectrally dispersed PL onto a silicon iCCD (PI-MAX4, Princeton Instruments). The sample was mounted in a gas-exchange helium cryostat (Oxford Instruments, OptistatCF2) which allowed variation of the temperature from 4 to 295 K in increments of between 5 and 50 K.

2.3. Time-correlated single-photon counting (TCSPC)

To measure the time-resolved PL of $Cs_2AgBiBr_6$ thin films, the sample was photoexcited by a 398 nm picosecond pulsed diode laser (PicoHarp, LDH-D-C-405M). Pulsed excitation with a fluence of 240 nJ cm^{-2} was used. The resultant PL was collected and coupled into a grating spectrometer (Princeton Instruments, SP-2558), which directed the spectrally dispersed PL onto a photon-counting detector (PDM series from MPD). The sample was mounted in a gas-exchange helium cryostat (Oxford Instruments, OptistatCF2) which allowed variation of the temperature from 4 to 295 K in increments of between 5 and 50 K.

2.4. Optical pump terahertz probe (OPTP) spectroscopy

An amplified laser system (Spectra Physics, MaiTai - Empower - Spitfire) with a central wavelength of 800 nm, 35 fs pulse duration and 5 kHz repetition rate was used to generate THz radiation via the inverse spin Hall effect and was detected using free-space electro-optic sampling with a 1 mm-thick ZnTe (110) crystal, a Wollaston prism and a pair of balanced photodiodes. The THz pulse was measured in transmission geometry. The pump beam was frequency-doubled to 400 nm by a β-barium-borate (BBO) crystal.

The temperature-dependent measurements were carried out with the samples mounted in a cold-finger helium cryostat (Oxford Instruments, MicrostatHe). The temperature was regulated via a temperature controller (Oxford Instruments, MercuryiTC) connected to a resistive heater and temperature sensors mounted both on the cryostat heat exchanger and at the end of the sample holder. The OPTP setup used here has an instrument response function with σ_{G}= 290 fs.

2.5. Photoluminescence upconversion spectroscopy

Samples were loaded into a vacuum cell in the glovebox, then placed under vacuum with continuous pumping and pressure 3 x 10^{-3} mbar during measurements. For upconversion measurements a tunable Ti:Sapphire laser (Mai Tai, Spectra Physics) with 80 MHz repetition rate and 80 fs pulse length was used to generate excitation pulses, frequency doubled in a BBO crystal to 405 nm. Excitation power was attenuated to 28.5 mW using a half-waveplate and vertical polarizer, giving an excitation fluence of 0.77 µJ cm^{-2} with a beam area of 4.6 x 10^{-4} cm^{2}.

The experimental setup used for upconversion was as described previously[5–7] with photoluminescence from the sample focussed into a BBO crystal and overlapped with a vertically polarized gate beam. The time delay of the gate pulses relative to excitation was controlled using a delay stage, to give upconverted (sum frequency) signal only when both pulses were present in the crystal simultaneously. The signal beam was focussed into a grating monochromator (Triax, Horiba) through a variable-width entry slit, and dispersed signal was then detected with a nitrogen-cooled silicon charge-coupled device (Symphony, Horiba). Long-pass and bandpass filters reduced background by blocking the excitation beam before the BBO crystal, and any unaltered PL or gate wavelengths after the crystal, to facilitate selective detection of upconverted light. This setup gives time resolution of 410 fs.

3. Fits to the absorption spectra

3.1. Indirect and direct band gap fits

In the absence of Coulomb correlations, the absorption coefficient, α_i, of a semiconductor above an indirect band gap at E_g^i is theoretically expected[8–10] to have a quadratic dependence on energy E:

$$\alpha_i \propto \left(E - E_g^i\right)^2$$

Meanwhile, the absorption coefficient, α_d, of a semiconductor above a direct band gap at E_g^d is theoretically expected[8,9] to have a square-root dependence on energy E:

$$\alpha_d \propto \left(E - E_g^d\right)^{1/2}$$

As has previously been done for $MAPbI_3$[9] and $Cs_2AgBiBr_6$[2], we fit these relationships to our measured absorption spectra in order to estimate the indirect and direct bandgaps, as shown in Figure S1.

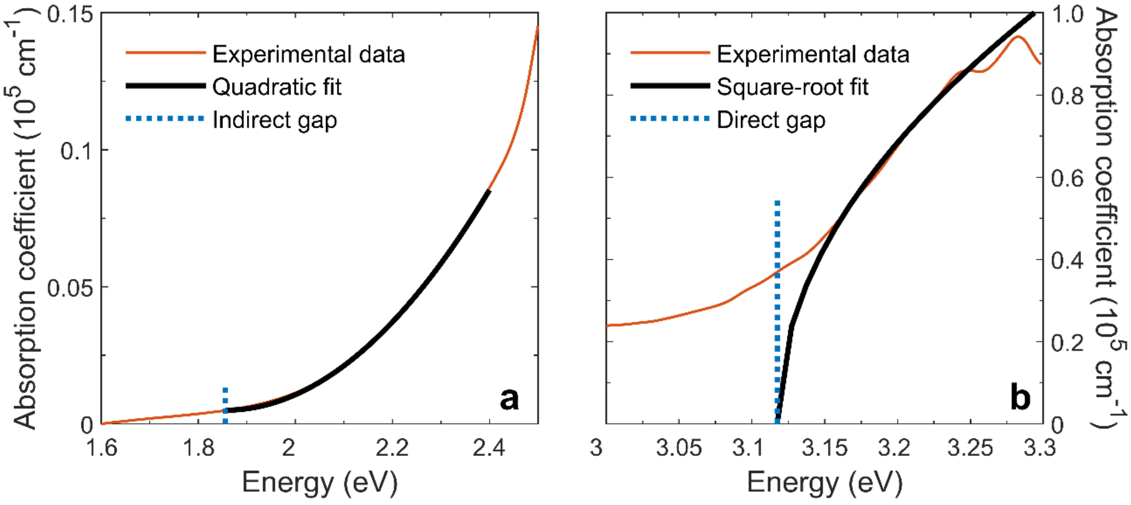

Figure S1. Close-up views of the absorption spectrum (red lines) of a $Cs_2AgBiBr_6$ thin film at 4K, in the energy ranges around the indirect band gap onset **(a)** and direct gap onset **(b)**. The respective quadratic and square-root fits to the onsets are plotted as solid black lines, while the estimates of the indirect and direct gap energies from these fits are indicated by dotted blue lines. The full spectrum is shown in Figure 1a of the main text.

We note that it is from these relationships that the Tauc technique[11] of determining the bandgap is derived, which has often been used to estimate the indirect[1,3,4,12–16] and direct[4,12,13,15,17] band gaps of $Cs_2AgBiBr_6$ by extrapolating from a linear fit to logarithmic absorption data. Zanatta[10] found that the Tauc method tended to produce very similar estimates of band gaps to fitting the above expressions for the absorption coefficient directly to the data, and indeed our estimate of E_g^i = 1.9 eV at 295 K is consistent with the values previously obtained from Tauc plots. [1,3,4,12–16] Using Tauc plots has however tended to underestimate[4,13,17] the direct band gap of $Cs_2AgBiBr_6$ (at between 2.2- 2.41 eV,

rather than the 3.03 eV we obtain at room temperature), perhaps because the excitonic onset was not accounted for or was mistaken for the direct gap onset. [18]

3.2. Elliott fitting

Alternatively, in a more complex approach that accounts for Coulomb correlations, the absorption spectrum of $Cs_2AgBiBr_6$ at 295 K was fitted at the direct band edge with an expression based on Elliott's theory, [19] which describes the absorption of a semiconductor near the band edge. Here, we follow the treatment described by Wright *et al.*, [20] in which the energy (E) dependent absorption, $\alpha(E, E_g, E_b)$, is described by a combination of contributions from bound excitons and electron-hole continuum states, involving the exciton binding energy E_b and band gap energy E_g:

$$\alpha(E, E_g, E_b) = \frac{b_0}{E}\sum_{n=1}^{\infty}\frac{4\pi E_b^{3/2}}{n^3}\delta\left(E - \left[E_g - \frac{E_b}{n^2}\right]\right) + \frac{b_0}{E}\left[\frac{2\pi\sqrt{\frac{E_b}{E - E_g}}}{1 - \exp\left(-2\pi\sqrt{\frac{E_b}{E - E_g}}\right)}\right] c_0^{-1}\,\mathrm{JDoS}(E)$$

where the joint density of states is given by $\mathrm{JDoS}(E) = c_0\sqrt{E - E_g}$ for $E > E_g$, and 0 otherwise, and the joint density of states constant $c_0 = \frac{1}{(2\pi)^2}\left(\frac{2\mu}{\hbar^2}\right)^{3/2} \times 2$, where μ is the reduced effective mass of the electron-hole system. The first term in the equation contains the contribution from bound excitonic states, while the second term contains the contributions from the continuum states. Broadening due to electron-phonon interactions, local fluctuations in the stoichiometry of the material, and energetic disorder is represented by convolution of $\alpha(E)$ with a normal distribution $\mathcal{N}(0, \Gamma^2)$ which has mean 0 and standard deviation Γ.

The final Elliott function, $f_{\mathrm{Elliott}} = \alpha(E, E_g, E_b) \otimes \mathcal{N}(0, \Gamma^2)$, (where convolution is represented by the symbol ⊗) was fitted to the absorption onset by a least-squares minimization method. The fit (black dashed line) to the experimental data at 295 K (red line) is shown in Figure S2, along with its constituent contributions from the continuum states (blue dotted line) and excitonic states (green dotted line). We obtained a direct band gap value of E_g = 3.059±0.001 eV, exciton binding energy value of E_b = 244±2 meV and a Γ value of 75±6 meV, corresponding to a full width at half-maximum (FWHM) excitonic linewidth of 176±14 meV. These values are in good agreement with those obtained by our alternative fitting methods (see Figure 2 of the main text), and previous Elliott fitting performed on the absorption spectrum of $Cs_2AgBiBr_6$. [15] However, we note that Elliott's theory assumes that the material has weakly bound Wannier excitons, [15] which may explain our difficulty in performing Elliott fitting on the lower temperature spectra, when the exciton binding energy was even larger. The small degree of overlap between the continuum and excitonic contributions justifies our approach in the main text of extracting the exciton energy from the center of a Gaussian fit to the excitonic absorption peak, and separate fitting to find the direct band gap energy as detailed in Section 3.1. above.

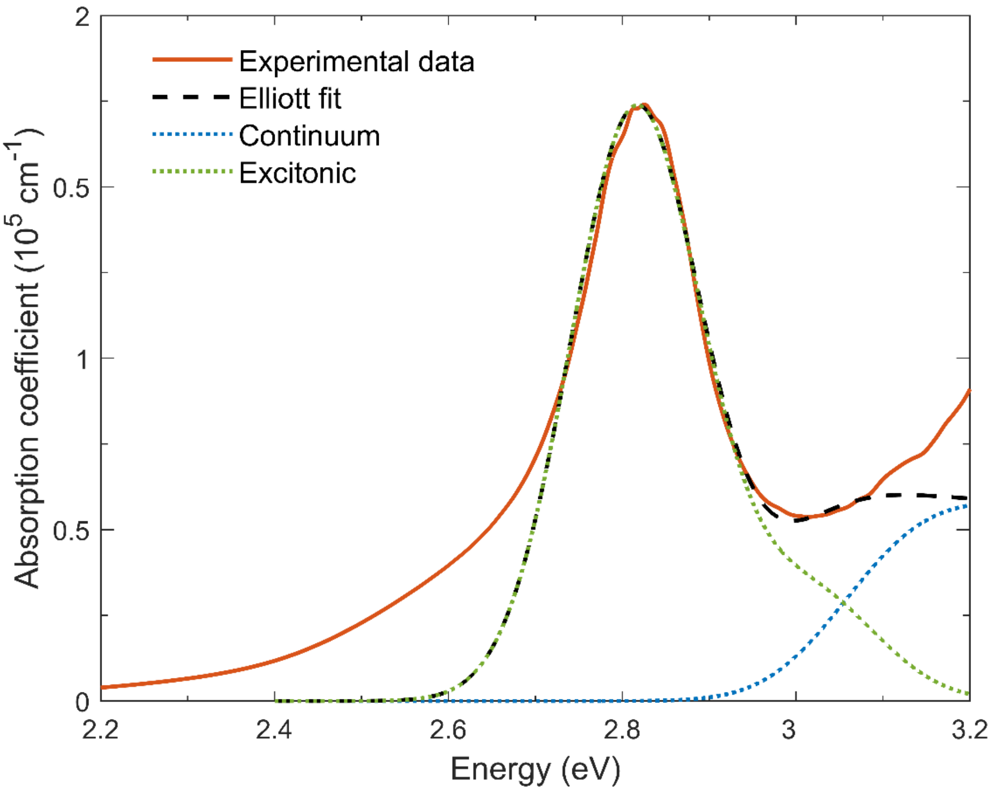

Figure S2. Elliott fit to the absorption spectrum of a $Cs_2AgBiBr_6$ thin film at 295 K. The experimental data are plotted in red, with the Elliott fit shown as a black dashed line. The contributions to the Elliott fit from the continuum and excitonic states are respectively plotted as blue and green dotted lines. The Elliott fit parameters used were E_g = 3.059 eV, E_b = 244 meV and Γ = 75 meV.

4. Temperature-dependent TCSPC measurements

The PL transients of a $Cs_2AgBiBr_6$ thin film from 4 to 295 K, measured using TCSPC at the wavelength of maximum PL intensity, are shown in Figure S3. The behaviour of the transient decays was very similar to that reported by Schade *et al.*[2] for a $Cs_2AgBiBr_6$ single crystal, suggesting that charge-carrier recombination in this material is dominated by intrinsic factors rather than depending on the processing method.

At temperatures from 4 K up to that of the structural phase transition at $T_S \approx 122$ K, the transients all exhibit an initial decay with a lifetime of approximately 1 ns, followed by one whose lifetime is much longer at the lowest temperatures (greater than 10 µs) but which speeds up with increasing temperature, as shown in Figure S3a. At higher temperature (in the cubic phase), the transients are smoothly curved and decay more rapidly with increasing temperature, as shown in Figure S3b, until their lifetimes are of the order of 1 ns close to room temperature. Following Schade *et al.*,[2] we understand the trends in the temperature-dependence of the PL transients in terms of two PL decay channels: a short channel (‘*S*’) present in the low-temperature tetragonal phase, and a longer channel (‘*L*’) present throughout. The former PL decay channel is governed by charge trapping at twinning defects, which are only present in the low-temperature tetragonal phase.[2] Both PL decay channels represent processes which are distinct from, and slower than, the ultrafast localization of charge carriers observed in our PL upconversion and OPTP photoconductivity transients, as shown in Figure 3 of the main text.

By modelling *S* as monoexponential, and *L* with a stretched exponential, it was possible to fit the PL transients faithfully across the entire temperature range, and to extract lifetimes for the PL decay components, as shown in Figure S3c. The stretched exponential function had the form $I = I_0 \exp\left(-(t/\tau_{\mathrm{c}})^{\beta}\right)$, where β is the distribution coefficient and τ_{c} is the characteristic lifetime: the time taken for the PL intensity to drop to I_0/e. [21,22] Since the distribution coefficients of the stretched exponentials could be as low as 0.14, the usual expression for calculating the average lifetime ($\tau_{\mathrm{av}} = \frac{\tau_c}{\beta}\Gamma\left(\frac{1}{\beta}\right)$) of a stretched exponential function is not a very meaningful average, [22] so the characteristic lifetime τ_{c} was used instead. In Section 5.1 below, the temperature-dependent lifetime of the *L*-channel decay is used to estimate the rate of charge-carrier recombination from the localized state which follows the ultrafast localization.

The decay of *L* becomes faster on warming, as is typical for metal halide perovskites, [23] though it remains much slower than that of the short component *S*, while its overall intensity decreases. In the low-temperature tetragonal phase, the contribution of *S* to the fits rapidly becomes ineffective as the tetragonal distortion significantly reduces with increasing temperature. At room temperature, the short PL lifetimes may appear at odds with the long carrier lifetimes on the order of 1 µs for which $Cs_2AgBiBr_6$ has been lauded. [13,24] However, these lifetimes were derived from a very low-intensity tail, which has been ascribed to recombination of charges in shallow trap states[14] and is in any case likely to be an independent PL decay channel from those considered here. [2] A comparably short PL lifetime to that we obtain at room temperature has also been reported by Longo *et al.*[1] from fitting to the initial part of the decay at room temperature.

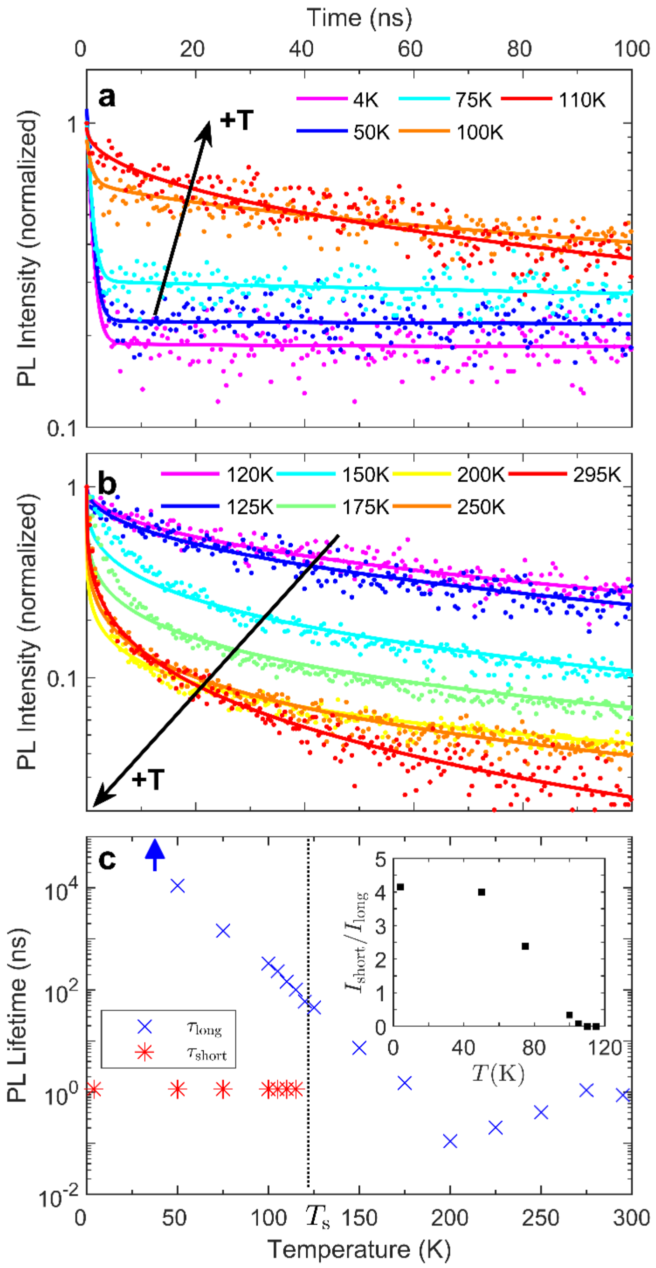

Figure S3. PL transients measured at the wavelength of maximum PL intensity for a $Cs_2AgBiBr_6$ thin film at temperatures between 4 and 110 K (tetragonal phase) are plotted as dots in **(a)**. Global fits of the sum of a stretched exponential (long) and a monoexponential (short) component, with the lifetime of the latter common between the temperatures (following the treatment of Schade *et al.*[2]), are shown as solid lines. PL transients at temperatures from 120 to 295 K (cubic phase) are shown as dots in **(b)**, with the lines now representing solely stretched exponential fits. The overall temperature trend of the shapes of the transients in both phases are indicated by black arrows. The lifetimes of the long (blue) and short (red) components are shown in **(c)**, on both sides of the phase transition temperature indicated by the dotted line labelled T_s. Below 50 K, the fitted long component lifetimes exceed the measurement capability of the system, as indicated by the blue arrow. The inset shows the temperature dependence of the ratio between the intensities of the short and long components.

5. THz photoconductivity

5.1. Fitting of temperature-dependent decays

The optical-pump terahertz-probe (OPTP) decays in Figure 3a of the main text were fitted using a two-level model (depicted in Figure S4) to account for the effect of ultrafast localization of the charge carriers on their mobilities. Within the model, initial photoexcitation occurs to a delocalized state associated with a high mobility μ_{deloc} and population n_{deloc}. This state determines the photoconductivity response at *t* = 0 ps. Subsequently, the charge carriers may rapidly relax (i.e. undergo self-trapping) at rate k_{loc} from the delocalized photoexcited state to a localized state with population n_{loc} and mobility μ_{loc}, from which they recombine over longer time scales at rate k_1 to the ground state.

Recombination from the localized state was observed in the time-resolved PL shown in Figure S3. Following the convention of Richter *et al.*, [25] the carrier lifetime is twice the PL lifetime, so $k_1 = \frac{1}{2\tau_{\mathrm{long}}}$, where τ_{long} is lifetime of the long component (‘*L*’) of the PL transient, obtained from the characteristic lifetime of stretched exponential fits and plotted in Figure S3c.

The initial charge carrier density was assumed to all be in the delocalized state, and was calculated to be $n_{\mathrm{deloc}}(t = 0) = 0.513 \times 10^{18}$ cm^{-3} from knowledge of the absorbed photon density profile.

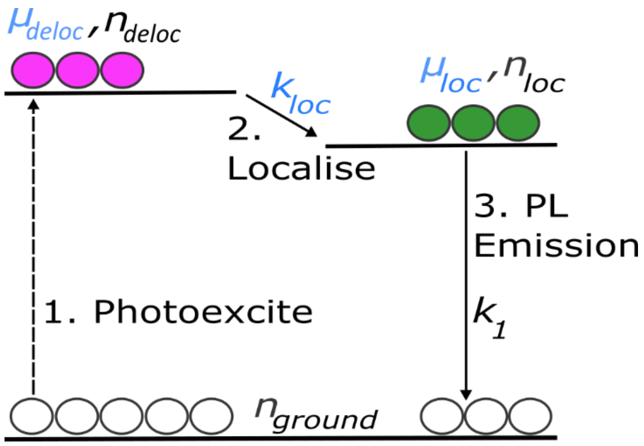

Figure S4. Schematic of the two-level mobility model used to fit the OPTP decays in Figure 3a of the main text. The fixed parameters are shown in black, while parameters that are fitted and extracted from the model are shown in blue. After being initially photoexcited to the delocalized state (pink), the charge-carriers localize to the localized state (green) before recombining.

Using the simplifying assumption that only the transitions depicted in Figure S4 occur, the time evolution of the populations of the states can be described by the following rate equations:

$$\frac{\partial n_{\mathrm{deloc}}}{\partial t} = -k_{\mathrm{loc}} n_{\mathrm{deloc}}$$

$$\frac{\partial n_{\mathrm{loc}}}{\partial t} = -k_1 n_{\mathrm{loc}} + k_{\mathrm{loc}} n_{\mathrm{deloc}}$$

which have the solutions

$$n_{\mathrm{deloc}}(t) = n_0 e^{-(k_{\mathrm{loc}})t}$$

$$n_{\mathrm{loc}}(t) = n_0 \frac{k_{\mathrm{loc}}}{k_{\mathrm{loc}} - k_1}\left(e^{-k_1 t} - e^{-(k_{\mathrm{loc}})t}\right)$$

describing the time-evolution of the charge-carrier volume density in each state. These may be related to the measured relative photoinduced THz transmission change $\Delta T/T$ via the sheet photoconductivity to which the population and mobility of both states contribute such that

$$\Delta\sigma = e(n_{\mathrm{deloc}}\mu_{\mathrm{deloc}} + n_{\mathrm{loc}}\mu_{\mathrm{loc}}),$$

assuming Drude-like charge-carrier transport. This assumption is supported by our measurements of the OPTP THz photoconductivity spectra of a $Cs_2AgBiBr_6$ thin film. As shown in Figure S6, the real and imaginary parts of the spectra are both flat, with the imaginary part near zero, which is consistent with Drude-like conductivity. Meanwhile, for a thin film on a thick quartz substrate, [26]

$$\Delta\sigma = -\frac{\epsilon_0 c(n_A + n_B)}{d_{\mathrm{film}}}\left(\frac{\Delta T}{T}\right)$$

where $n_A = 1$ is the refractive index of the vacuum, $n_B = 2.13$ is the refractive index of the z-cut quartz substrate, [27] and $d_{\mathrm{film}} = 290$ nm is the film thickness.

Therefore, the overall $\Delta T/T$ signals shown in Figure 3a of the main text can be described by the following expression derived from our model:

$$\frac{\Delta T}{T} = -\frac{n_0 e d_{\mathrm{film}}}{\epsilon_0 c(n_A + n_B)}\left(\left[\mu_{\mathrm{deloc}} - \frac{\mu_{\mathrm{loc}} k_{\mathrm{loc}}}{k_{\mathrm{loc}} - k_1}\right] e^{-(k_{\mathrm{loc}})t} + \frac{\mu_{\mathrm{loc}} k_{\mathrm{loc}}}{k_{\mathrm{loc}} - k_1} e^{-k_1 t}\right)$$

Finally, in order to account for the Instrument Response Function of our OPTP system this expression was convolved with a normalized Gaussian pulse with $\sigma_{\mathrm{G}} = 290$ ps arriving at time t_0. The overall expression that was fitted to the data, including the offset in time t_0, is thus as follows:

$$\frac{\Delta T}{T} = -\frac{n_0 e d_{\mathrm{film}}}{2\epsilon_0 c(n_A + n_B)}\left(\left[\mu_{\mathrm{deloc}} - \frac{\mu_{\mathrm{loc}} k_{\mathrm{loc}}}{k_{\mathrm{loc}} - k_1}\right] e^{-(k_{\mathrm{loc}})(t-t_0)+\left(\frac{k_{loc}\sigma_{\mathrm{G}}}{\sqrt{2}}\right)^2} \mathrm{Erfc}\left(\frac{k_{loc}\sigma_{\mathrm{G}}^2 - (t-t_0)}{\sigma_{\mathrm{G}}\sqrt{2}}\right) + \frac{\mu_{\mathrm{loc}} k_{\mathrm{loc}}}{k_{\mathrm{loc}} - k_1} e^{-k_1(t-t_0)+\left(\frac{k_1\sigma_{\mathrm{G}}}{\sqrt{2}}\right)^2} \mathrm{Erfc}\left(\frac{k_1\sigma_{\mathrm{G}}^2 - (t-t_0)}{\sigma_{\mathrm{G}}\sqrt{2}}\right)\right)$$

Here $\mathrm{Erfc}(x)$ is the complementary error function, defined as:

$$\mathrm{Erfc}(x) = \frac{2}{\sqrt{\pi}}\int_x^{\infty} e^{-y^2} dy$$

From these fits, values of μ_{loc}, μ_{deloc} and k_{loc}were extracted at each temperature, as shown in Figure 4a and Figure 4b of the main text.

5.2. Fluence-dependent OPTP THz photoconductivity spectroscopy transients

Fluence-dependent OPTP transients were measured out to 1000 ps at 295 K and 4 K using logarithmically spaced points in time, as shown in Figure S5 panels a and c respectively. Since $-\Delta T/T$ is directly proportional to the photoconductivity, its value correlates with the charge-carrier mobility of the system. A fast decay in $-\Delta T/T$ is evident at short times at both temperatures, with its lack of fluence dependence evident from panels b and d, in which the normalized transients are plotted. The fast decay is much more dominant at 4 K, when the difference in the mobilities of the delocalized and localized states, which contribute to the photoconductivity more prominently at short and long times respectively, is at its largest.

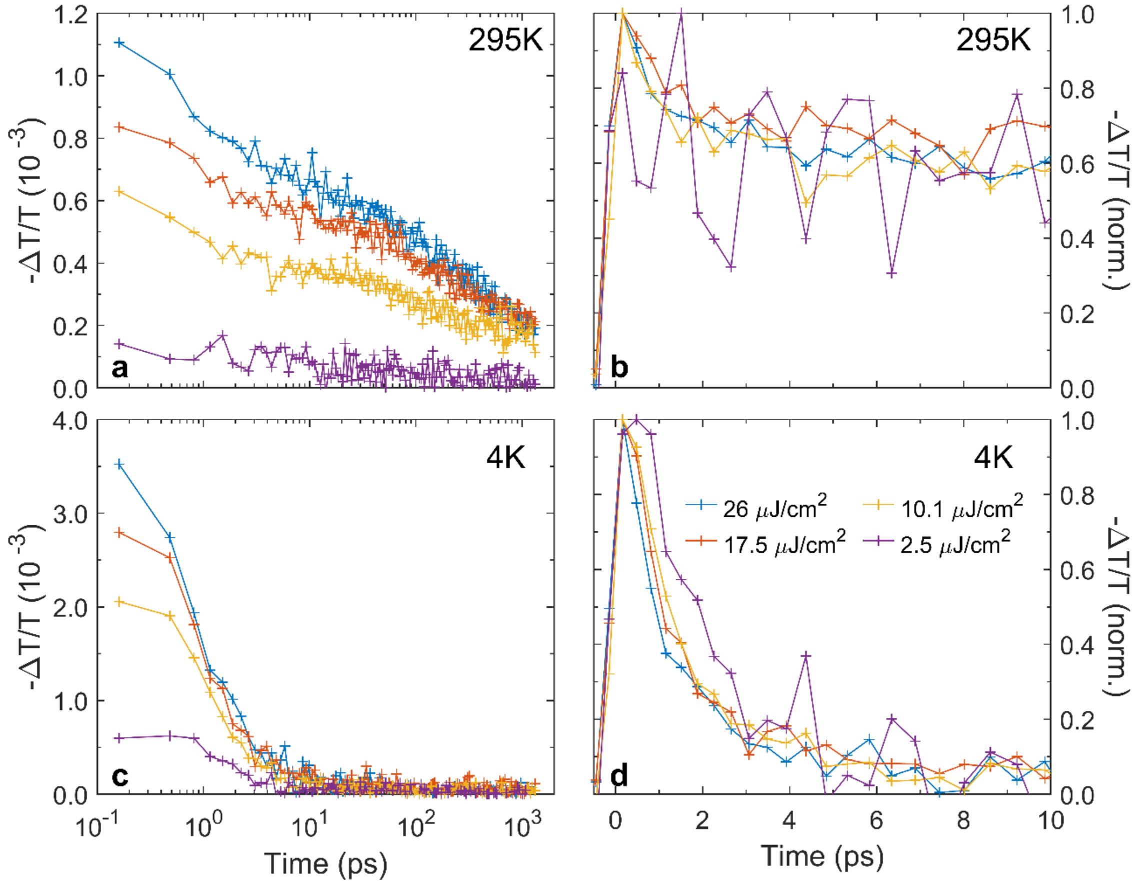

Figure S5. (a) Fluence-dependent OPTP THz photoconductivity transients of a $Cs_2AgBiBr_6$ thin film on a logarithmic time axis at 295 K, shown normalized and against a linear time axis in **(b)**. The corresponding transients at 4 K are shown in **(c)** on a logarithmic time axis, while **(d)** shows these data normalized and on a linear time axis. Data taken for four excitation fluences is presented in each panel, as defined in the legend in **(d)**.

5.3. OPTP THz photoconductivity spectra

The OPTP THz photoconductivity spectra were measured at room temperature at time delays of approximately 20 and 200 ps at a fluence of 2 μJcm^{-2} following excitation at 400 nm in low vacuum (< 10^{-2} mbar), and the photoconductivity was calculated in the same way as Section 5.1 above as:

$$\Delta\sigma(\omega) = -\frac{\epsilon_0 c(n_A + n_B)}{d_{\text{film}}}\left(\frac{\Delta T(\omega)}{T(\omega)}\right)$$

The real and imaginary parts are plotted in Figure S6, and show flat photoconductivity responses across short and long time delays, with no peaks or resonances across the measured frequency range, consistent with our above assumption of Drude-like carrier transport. Given that OPTP spectroscopy measures couplings between photoexcited charge carriers and local electric fields across the energy range of 0.5 – 10 meV, these spectra help us to rule out localization states or intra-excitonic transitions at these energies. This is in good agreement with the large exciton binding energy (E_b) measured here (see Figure 2a of the main text), of 212 meV at room temperature, which (assuming a simple hydrogenic binding model) gives a transition energy of $\frac{3}{4}E_b \sim 160$ meV to go from the n = 1 to 2 excitonic states, well outside of the range being probed here.

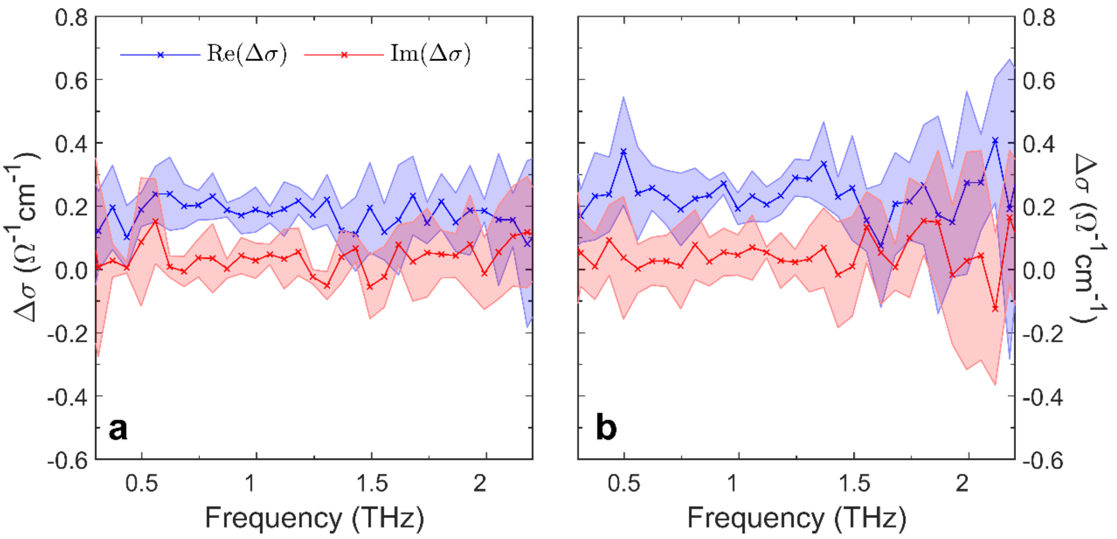

Figure S6. OPTP THz photoconductivity spectra measured on a $Cs_2AgBiBr_6$ thin film at delays of **(a)** 20 ps and **(b)** 200 ps at 295 K. The blue lines indicate the real part of the change in photoconductivity (*Δσ*) while the red lines indicate the imaginary part. The blue and red shaded regions show the errors in the correspondingly colored data points.

5.4. OPTP THz dark conductivity spectra

The dark conductivity was measured at room temperature in low vacuum (< 10^{-2} mbar), and the response in the terahertz frequency regime can be calculated as[26,28]

$$\sigma(\omega) = \frac{\epsilon_0 c(n_A + n_B)}{d_{\text{film}}}\left(\frac{T_{\text{ref}}(\omega)}{T_{\text{sample}}(\omega)} - 1\right)$$

where $T_{\mathrm{sample}}, T_{\mathrm{ref}}$ are the transmitted terahertz intensity for the $Cs_2AgBiBr_6$ sample, and a quartz substrate, respectively.

The real part of the dark conductivity spectrum shows a clear peak at approximately 1.7 THz (~57 cm^{-1}). The phonon modes calculated by Steele *et al.*[4] at 0 K for the $Cs_2AgBiBr_6$ lattice give several modes clustered around 1.5 THz (50 cm^{-1}), associated with the Ag-Br and Bi-Br ionic sublattices, in good agreement with the peak observed here.

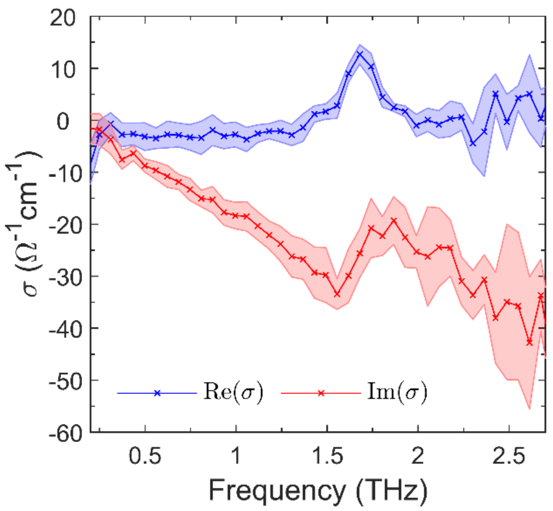

Figure S7. OPTP THz dark conductivity spectra measured on a $Cs_2AgBiBr_6$ thin film at 295 K. The blue line indicates the real part of the conductivity (σ) while the red line indicates the imaginary part. The blue and red shaded regions represent the errors in the correspondingly colored data points.

References

(1) Longo, G.; Mahesh, S.; Buizza, L. R. V.; Wright, A. D.; Ramadan, A. J.; Abdi-Jalebi, M.; Nayak, P. K.; Herz, L. M.; Snaith, H. J. Understanding the Performance-Limiting Factors of $Cs_2AgBiBr_6$ Double-Perovskite Solar Cells. *ACS Energy Lett.* **2020**, *5*, 2200–2207.

(2) Schade, L.; Wright, A. D.; Johnson, R. D.; Dollmann, M.; Wenger, B.; Nayak, P. K.; Prabhakaran, D.; Herz, L. M.; Nicholas, R.; Snaith, H. J.; Radaelli, P. G. Structural and Optical Properties of $Cs_2AgBiBr_6$ Double Perovskite. *ACS Energy Lett.* **2019**, *4*, 299–305.

(3) Zelewski, S.; Urban, J.; Surrente, A.; Maude, D. K.; Kuc, A. B.; Schade, L.; Johnson, R. D.; Dollmann, M.; Nayak, P.; Snaith, H.; Radelli, P.; Kudrawiec, R.; Nicholas, R. J.; Plochocka, P.; Baranowski, M. Revealing the Nature of Photoluminescence Emission in the Metal-Halide Double Perovskite $Cs_2AgBiBr_6$. *J. Mater. Chem. C* **2019**, *7*, 8350–8356.

(4) Steele, J. A.; Puech, P.; Keshavarz, M.; Yang, R.; Banerjee, S.; Debroye, E.; Kim, C. W.; Yuan, H.; Heo, N. H.; Vanacken, J.; Walsh, A.; Hofkens, J.; Roeffaers, M. B. J. Giant Electron-Phonon Coupling and Deep Conduction Band Resonance in Metal Halide Double Perovskite. *ACS Nano* **2018**, *12*, 8081–8090.

(5) Savill, K. J.; Klug, M. T.; Milot, R. L.; Snaith, H. J.; Herz, L. M. Charge-Carrier Cooling and Polarization Memory Loss in Formamidinium Tin Triiodide. *J. Phys. Chem. Lett.* **2019**, *10*, 6038–6047.

(6) Chang, M. H.; Hoeben, F. J. M.; Jonkheijm, P.; Schenning, A. P. H. J.; Meijer, E. W.; Silva, C.; Herz, L. M. Influence of Mesoscopic Ordering on the Photoexcitation Transfer Dynamics in Supramolecular Assemblies of Oligo-*p*-Phenylenevinylene. *Chem. Phys. Lett.* **2006**, *418*, 196–201.

(7) Chang, M. H.; Hoffmann, M.; Anderson, H. L.; Herz, L. M. Dynamics of Excited-State Conformational Relaxation and Electronic Delocalization in Conjugated Porphyrin Oligomers. *J. Am. Chem. Soc.* **2008**, *130*, 10171–10178.

(8) Yu, P. Y.; Cardona, M. *Fundamentals of Semiconductors*; Springer-Verlag: Berlin Heidelberg, 2010.

(9) Kirchartz, T.; Rau, U. Decreasing Radiative Recombination Coefficients via an Indirect Band Gap in Lead Halide Perovskites. *J. Phys. Chem. Lett.* **2017**, *8*, 1265–1271.

(10) Zanatta, A. R. Revisiting the Optical Bandgap of Semiconductors and the Proposal of a Unified Methodology to Its Determination. *Sci. Rep.* **2019**, *9*, 11225.

(11) Tauc, J.; Grigorovici, R.; Vancu, A. Optical Properties and Electronic Structure of Amorphous Germanium. *Phys. Stat. Sol.* **1966**, *15*, 627–637.

(12) Bekenstein, Y.; Dahl, J. C.; Huang, J.; Osowiecki, W. T.; Swabeck, J. K.; Chan, E. M.; Yang, P.; Alivisatos, A. P. The Making and Breaking of Lead-Free Double Perovskite Nanocrystals of Cesium Silver-Bismuth Halide Compositions. *Nano Lett.* **2018**, *18*, 3502–3508.

(13) Slavney, A. H.; Hu, T.; Lindenberg, A. M.; Karunadasa, H. I. A Bismuth-Halide Double Perovskite with Long Carrier Recombination Lifetime for Photovoltaic Applications. *J. Am. Chem. Soc.* **2016**, *138*, 2138–2141.

(14) Bartesaghi, D.; Slavney, A. H.; Gélvez-Rueda, M. C.; Connor, B. A.; Grozema, F. C.; Karunadasa, H. I.; Savenije, T. J. Charge Carrier Dynamics in $Cs_2AgBiBr_6$ Double Perovskite. *J. Phys. Chem. C* **2018**, *122*, 4809–4816.

(15) Kentsch, R.; Scholz, M.; Horn, J.; Schlettwein, D.; Oum, K.; Lenzer, T. Exciton Dynamics and Electron-Phonon Coupling Affect the Photovoltaic Performance of the $Cs_2AgBiBr_6$ Double Perovskite. *J. Phys. Chem. C* **2018**, *122*, 25940–25947.

(16) McClure, E. T.; Ball, M. R.; Windl, W.; Woodward, P. M. Cs_2AgBiX_6 (X = Br, Cl): New Visible Light Absorbing, Lead-Free Halide Perovskite Semiconductors. *Chem. Mater.* **2016**, *28*, 1348–1354.

(17) Pantaler, M.; Cho, K. T.; Queloz, V. I. E.; García Benito, I.; Fettkenhauer, C.; Anusca, I.; Nazeeruddin, M. K.; Lupascu, D. C.; Grancini, G. Hysteresis-Free Lead-Free Double-Perovskite Solar Cells by Interface Engineering. *ACS Energy Lett.* **2018**, *3*, 1781–1786.

(18) Parrott, E. S.; Green, T.; Milot, R. L.; Johnston, M. B.; Snaith, H. J.; Herz, L. M. Interplay of Structural and Optoelectronic Properties in Formamidinium Mixed Tin–Lead Triiodide Perovskites. *Adv. Funct. Mater.* **2018**, *28*, 1802803.

(19) Elliott, R. J. Intensity of Optical Absorption by Excitions. *Phys. Rev.* **1957**, *108*, 1384–1389.

(20) Wright, A. D.; Volonakis, G.; Borchert, J.; Davies, C. L.; Giustino, F.; Johnston, M. B.; Herz, L. M. Intrinsic Quantum Confinement in Formamidinium Lead Triiodide Perovskite. *Nat. Mater.* **2020**, *19*, 1201–1206.

(21) Lindsey, C. P.; Patterson, G. D. Detailed Comparison of the Williams–Watts and Cole–Davidson Functions. *J. Chem. Phys.* **1980**, *73*, 3348–3357.

(22) Johnston, D. C. Stretched Exponential Relaxation Arising from a Continuous Sum of Exponential Decays. *Phys. Rev. B* **2006**, *74*, 184430.

(23) Milot, R. L.; Eperon, G. E.; Snaith, H. J.; Johnston, M. B.; Herz, L. M. Temperature-Dependent Charge-Carrier Dynamics in $CH_3NH_3PbI_3$ Perovskite Thin Films. *Adv. Funct. Mater.* **2015**, *25*, 6218–6227.

(24) Hoye, R. L. Z.; Eyre, L.; Wei, F.; Brivio, F.; Sadhanala, A.; Sun, S.; Li, W.; Zhang, K. H. L.; MacManus-Driscoll, J. L.; Bristowe, P. D.; Friend, R. H.; Cheetham, A. K.; Deschler, F. Fundamental Carrier Lifetime Exceeding 1 µs in $Cs_2AgBiBr_6$ Double Perovskite. *Adv. Mater. Interfaces* **2018**, *5*, 1800464.

(25) Richter, J. M.; Abdi-Jalebi, M.; Sadhanala, A.; Tabachnyk, M.; Rivett, J. P. H.; Pazos-Outón, L. M.; Gödel, K. C.; Price, M.; Deschler, F.; Friend, R. H. Enhancing Photoluminescence Yields in Lead Halide Perovskites by Photon Recycling and Light Out-Coupling. *Nat. Commun.* **2016**, *7*, 13941.

(26) Ulatowski, A. M.; Herz, L. M.; Johnston, M. B. Terahertz Conductivity Analysis for Highly Doped Thin-Film Semiconductors. *J. Infrared Millim. Terahertz Waves* **2020**, *41*, 1431–1449.

(27) Davies, C. L.; Patel, J. B.; Xia, C. Q.; Herz, L. M.; Johnston, M. B. Temperature-Dependent Refractive Index of Quartz at Terahertz Frequencies. *J. Infrared Millim. Terahertz Waves* **2018**, *39*, 1236–1248.

(28) Joyce, H. J.; Boland, J. L.; Davies, C. L.; Baig, S. A.; Johnston, M. B. A Review of the Electrical Properties of Semiconductor Nanowires: Insights Gained from Terahertz Conductivity Spectroscopy. *Semicond. Sci. Technol.* **2016**, *31*, 103003.
